# Serum levels of vitamin A and 25-hydroxyvitamin D3 (25OHD3) as reflectors of pulmonary function and quality of life (QOL) in children with stable asthma: A case–control study

**DOI:** 10.1097/MD.0000000000009830

**Published:** 2018-02-16

**Authors:** Ya-Jie Bai, Ru-Jun Dai

**Affiliations:** a 5th Department of Pediatric; b 2nd Department of Pediatric, Cangzhou Central Hospital, Cangzhou, P.R. China.

**Keywords:** activity of daily living, 25-hydroxyvitamin D3, pulmonary function, quality of life, stable asthma, vitamin A

## Abstract

**Background:**

This study aims to explore the relationship between serum vitamin A and 25-hydroxyvitamin D3 (25OHD3) levels with pulmonary function and quality of life (QOL) in children with stable asthma.

**Methods:**

A total of 117 cases of children with stable asthma were assigned into the case group and 129 healthy children underwent physical examination during the same period into the control group. Electrochemiluminescence was employed to determine serum vitamin A and 25OHD3 levels. The children with stable asthma were further divided into the mild, moderate, and severe groups according to their degree of asthma. A pulmonary function meter was used to assess the pulmonary function indexes: percentage of forced expiratory volume in 1 sec/predictive value (FEV_1_%pred), forced vital capacity (FVC), forced expiratory volume in 1 sec/forced vital capacity (FEV_1_/FVC), peak expiratory flow (PEF), and maximal voluntary ventilation (MVV). The children's quality (QOL) of life with asthma was evaluated by their activities of daily living (ADLs) and Medical Research Council (MRC) scores. Pearson correlation analysis was applied to analyze the correlations of serum vitamin A and 25OHD3 levels with FEV_1_%pred, FVC, FEV_1_/FVC, PEF, MVV, ADL, and MRC.

**Results:**

Serum vitamin A and 25OHD3 levels were lower in children with stable asthma than those who were in the control group (*P* < .05). The severe group showed the lowest FEV_1_%pred, FVC, FEV_1_/FVC, PEF, MVV, and ADL scores, and the highest MRC score compared to the mild and moderate groups (all *P* < .05). Serum vitamin A and 25OHD3 levels were positively correlated with pulmonary function and ADL score in children with stable asthma, while serum vitamin A and 25OHD3 levels were negatively correlated with MRC score (all *P* < .05). In the case group, serum vitamin A and 25OHD3 levels were positively correlated with serum calcium and phosphorus levels (all *P* < .05).

**Conclusion:**

These findings indicate that increased serum vitamin A and 25OHD3 levels reflect good pulmonary function and good QOL in children with stable asthma.

## 1 Introduction

Bronchial asthma, or also known as asthma, is a very common respiratory disease, which often presents with clinical features such as airway infiltration of mast cells eosinophils and activated T helper lymphocytes.^[1]^ Asthma is a chronic inflammatory disease of the airways of the lungs that is found most frequently in the pediatric population.^[2]^ Asthma currently affects more than 0.3 billion in the world and is expected to affect another 0.1 billion people by 2025.^[1]^ The distribution of asthma in children is occurred most frequently in the western and is growing increasingly more common in other regions such as Central and South America, Eastern Europe, and Africa.^[3]^ Allergies are strongly linked to asthma and to other respiratory diseases such as sinusitis, ear infections, and nasal polyps. Asthma is regarded as an allergic condition, for which the risk factors that are involved with its onset include rhinovirus infections secondhand smoking and outdoor air pollution.^[3]^ Control is of pivotal importance in asthma management, before which identifying the severity of asthma must be included in the diagnosis. Long-term asthma management includes undergoing inhaled corticosteroids treatment in both children and adults.^[4]^ In addition, traditional Chinese medicine has been found to regulate the expression of leukotriene receptor gene and Th1/Th2 cellular immunity imbalance when children suffer from asthma.^[5]^ Current clinical trials have produced newer forms of treatments for allergy and immunology has been continuingly found in asthma therapy.^[6]^ Although many guidelines and medications are available, a definite cure for asthma remains to be found as well as a need for the control of asthmatic symptoms still remain inadequate.^[7]^

Vitamin A supplementation has been recently found to be helpful in asthma control.^[8]^ Vitamin A, also known as retinoic acid, is essential in normal development and differentiation of lung airways; furthermore, vitamin A deficiency is related to many lung problems including asthma.^[9]^ 25-Hydroxyvitamin D3 (25OHD3) can be obtained from various sources: the exposure of the epidermis to the sunlight allows the body to synthesize 25OHD3, we can take additional supplements of fish oil which contains high levels of 25OHD3.^[10]^ It has been found that lower serum 25OHD3 levels are epidemiologically related to higher occurrence of respiratory infections both in upper and lower respiratory tracts, and vitamin D deficiency is closely connected with badly aggravation of asthma.^[11]^ It has been demonstrated that 25OHD3 supplementation helps more to elevate quality of life (QOL) effectively than to alleviate the pulmonary damages caused by severe asthma.^[12]^ Therefore, we carry out this investigation in order to explore the effects of serum vitamin A and 25OHD3 on pulmonary function and QOL in children with asthma, in hopes of providing useful guidance for improving pulmonary function and QOL.

## 2 Materials and methods

### 2.1 Ethical statement

This study was approved by the Ethics Committee of Cangzhou Central Hospital. All children's parents had signed the informed consents regarding the procedures of the whole investigation.

### 2.2 Study subjects selection and criteria

From January 2013 to December 2016, 117 children with stable asthma were assigned into the case group, and 129 healthy children were assigned the control group during the same period after a thorough physical examination. Diagnostic criteria that was used to help assign children into the case group include^[13]^: scattered or diffuse wheeze mainly in expiratory phase can be heard in both lungs during an asthmatic attack with prolonged expiratory time, which could be treated or self-relief; recurrent episodes of wheezing, shortness of breath, chest tightness, or cough when exposed to cold air, allergens, chemical stimulation, physical stimulation, and upper respiratory tract virus infections, all of which could be treated or relieved by self. Inclusive criteria: patients are in accordance with the guidelines for prevention and treatment of bronchial asthma in China; patients are able to cooperate and comply with pulmonary function tests. Exclusion criteria: patients with restrictive ventilation dysfunction; patients with a previous history of calcium supplementation; patients who have recently taken vitamin D; patients with other serious diseases; and patients who are unable to complete the pulmonary function tests.

### 2.3 Blood collection and measurement

Three milliliter of the morning fasting peripheral venous blood was collected from children in the both case and control groups, respectively. Blood samples were placed in anticoagulation tubes containing ethylene diamine tetraacetic acid at room temperature for 60 minutes and then centrifuged for 10 minutes at 3000 rpm. The serum was kept at −80 °C. Serum vitamin A and 25OHD3 levels were measured by a full-automatic Chemiluminescence Immunoassay Analyzer (Roche Ccobas e601) with corollary reagents. The reaction was carried out using 2 steps: serum vitamin A and 25OHD3 in the serum samples first competed with biotin labeled vitamin A and 25OHD3, respectively. Immunocomplex was formed as a result through the biotin–streptavidin interaction after adding streptavidin labeled magnetic particle. ProCell was applied to wash away the materials that did not combine. Chemiluminescence was conducted after the electrode was added to the voltage. Luminous intensity was measured using a photomultiplier. All the steps were carried out strictly in accordance with the instructions.

### 2.4 Assessment and measurements of pulmonary function

Children in the case group and the control group were asked to be sat down and asked to put on a mouthpiece and nose clip. They were then asked to place their hands on the cheeks and raised their heads up with the neck straight and breathe normally. The pulmonary function indexes were recorded once the detector was stable which measured patients’ forced vital capacity (FVC), percentage of forced expiratory volume in 1 sec/predictive value (FEV_1_%pred), forced expiratory volume in 1 sec/forced vital capacity (FEV_1_/FVC), peak expiratory flow, and maximal voluntary ventilation. According to the criteria of pulmonary function, the levels were graded as^[14]^: mild, FEV_1_%pred ≥80% and FEV_1_/FVC (%) >70%; moderate, 60% ≤FEV_1_%pred <80% and FEV_1_/FVC (%) >70%; and severe, FEV_1_%pred <60% and FEV_1_/FVC (%) >70%. According to the different levels of pulmonary function, the case group was further subdivided into 3 subgroups: the mild group, the moderate group, and the severe group. After grading, the pulmonary function meter (Medikro Oy, Kuopio, Finland) was used to measure serum vitamin A/25OHD3 levels and pulmonary function indexes under different pulmonary functions.

### 2.5 QOL evaluation

QOL was evaluated based on the activity of daily living (ADL) score and Medical Research Council (MRC) score. ADL score standards^[15]^ were as follows: patients are unable to carry out their daily activities received a score of 1; patients who suffer severe pain during daily activities received a score of 2; patients who suffer moderate pain during daily activities received a score of 3; patients who suffer mild pain during daily activities received a score of 4; and patients do not suffer any form of pain during their daily activities received a score of 5. MRC scoring criteria were listed as the following^[16]^: patients who have severe breathing difficulties or have breathing difficulties when dressing or undressing themselves received a score of 4; patients who need to rest after walking for 100 m on a flat leveled platform received a score of 3; patients who walk slower than their peers on a flat bottom and need to rest after a long distance of walking received a score of 2; patients have breathing difficulties when walking quickly on a flat platform or a small slope received a score of 1; and patients whose wheezing occur only during intense exercises received a score of 0.

### 2.6 Statistical analysis

Statistical analysis was performed using SPSS21.0 software (SPSS Inc., Chicago, IL). Measurement data were presented as a mean ± standard deviation. Comparisons between the case and control groups were analyzed by a *t* test. Comparisons among multiple groups were carried out by an *F* test. Pearson correlation analysis was applied for analyzing correlation. Values of *P* < .05 were considered as statistically significant.

## 3 Results

### 3.1 Baseline characteristics of children in the case and control groups

Age, weight, and allergic history showed no significant differences in the 2 groups (all *P* > .05). The serum calcium level was 2.48 ± 0.22 mmol/L and the serum phosphorus level was 1.56 ± 0.29 mmol/L in the control group. The serum calcium level was 2.10 ± 0.47 mmol/L and the serum phosphorus level was 1.41 ± 0.24 mmol/L in the case group. The calcium and phosphorus levels in both groups showed significant differences between each other (all *P* < .05) (Table 1).

**Table 1:**
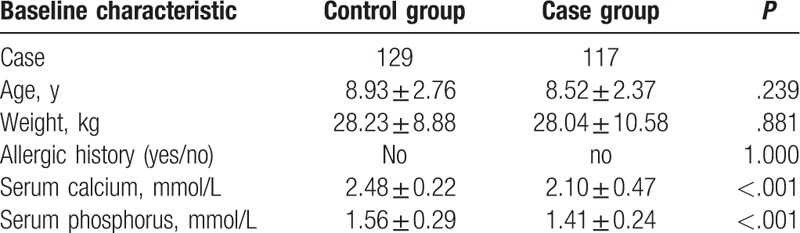
Comparisons of baseline characteristics of children in the case and control groups.

### 3.2 The severe group shows the lowest serum vitamin A and 25OHD3 levels

**Figure 1:**
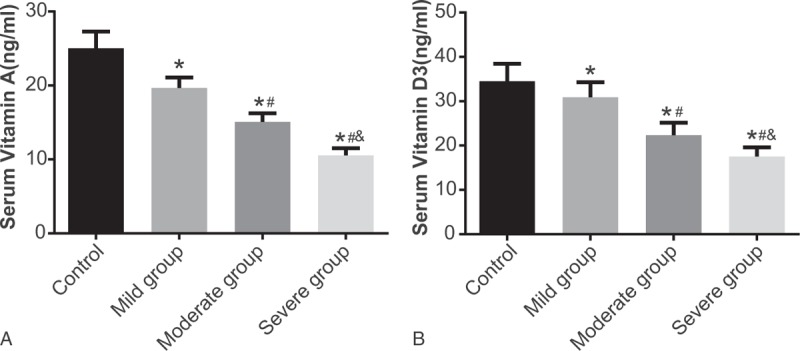
Serum vitamin A and 25OHD3 levels between the case group and the control group. Notes: ^∗^, compared with the control group, *P* < .05; ^#^, compared with the mild group, *P* < .05; and ^&^, compared with the severe group, *P* < .05; 25OHD3 = 25-hydroxyvitamin D3.

Electrochemiluminescence was employed to determine serum vitamin A and 25OHD3 levels. The results showed that serum vitamin A and 25OHD3 levels in 3 case groups were all lower than those in the control group (*P* < .05). The moderate and severe case groups showed lower serum vitamin A and 25OHD3 levels compared to the mild group. Additionally, the severe group showed lower serum vitamin A and 25OHD3 levels compared to the moderate group (Fig. 1).

### 3.3 Serum vitamin A and 25OHD3 levels are positively correlated with serum calcium and phosphorus levels

**Figure 2:**
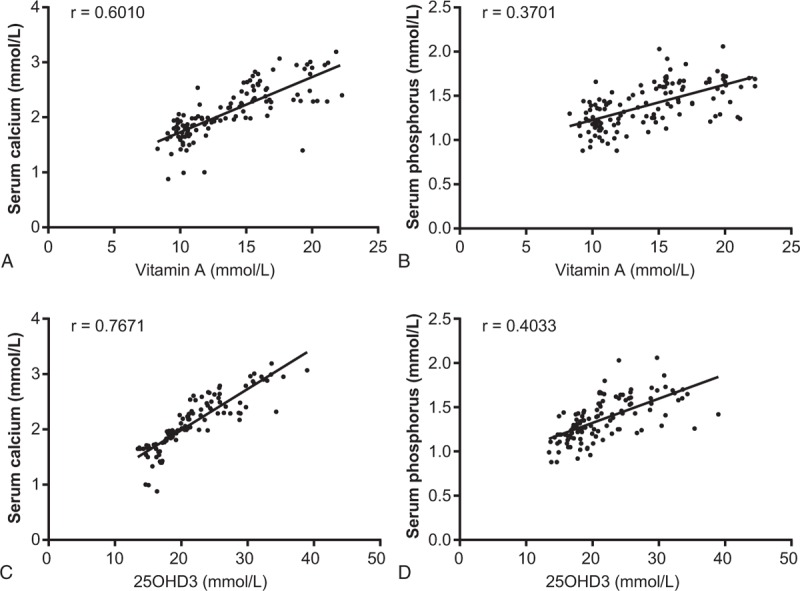
Serum vitamin A and 25OHD3 levels with serum calcium and phosphorus levels. (A) Correlation between serum vitamin A and serum calcium levels; (B) correlation between serum vitamin A and serum phosphorus levels; (C) correlation between 25OHD3 and serum calcium levels; and (D) correlation between 25OHD3 and serum phosphorus levels. 25OHD3 = 25-hydroxyvitamin D3.

In the case group, serum vitamin A and 25OHD3 levels were decreased, and serum calcium and phosphorus levels were also decreased in comparison to the control group. Correlation analysis revealed that serum vitamin A and 25OHD3 levels were positively correlated with serum calcium and phosphorus levels (all *P* < .05) (Fig. 2).

### 3.4 Serum vitamin A and 25OHD3 levels decreased differently according to the degree of asthma in children with stable asthma

According to the pulmonary function grading, patients in the case group were subdivided into 3 subgroups: the mild group (n = 21, FEV_1_%pred ≥80%, FEV_1_/FVC [%] >70%), the moderate group (n = 39, 60% ≤FEV_1_%pred <80%, FEV_1_/FVC [%] >70%), and the severe group (n = 57, FEV_1_%pred <60%, FEV_1_/FVC [%] >70%). Pulmonary function grading results showed that the serum vitamin A and 25OHD3 levels decreased differently according to the degree of asthma in children with stable asthma (*P* < .05) (Table 2).

**Table 2:**
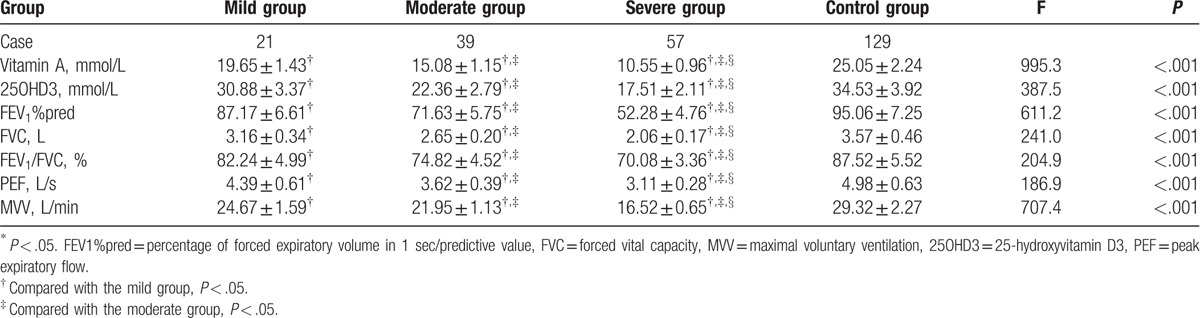
Comparisons of serum vitamin A and 25OHD3 levels with pulmonary function indexes in the case and control groups.

### 3.5 Children in the severe group have the lowest QOL

The results of the MRC and ADL scores demonstrated that the QOL in children with stable asthma in the mild, moderate, and severe groups decreased significantly compared to those in the control group (all *P* < .05). Compared with the mild and moderate groups, children in the severe group had the lowest QOL whereas those in the moderate group the 2nd highest and those in the mild group had the highest QOL (all *P* < .05) (Table 3).

**Table 3:**
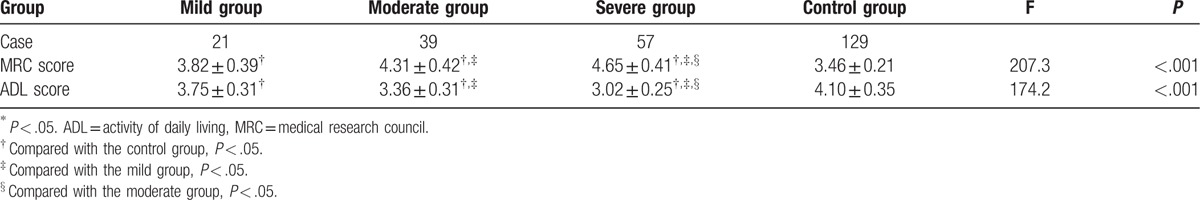
Comparisons of MRC and ADL scores in the case and control groups.

### 3.6 Correlations of the serum vitamin A and 25OHD3 levels with the pulmonary function and QOL in children with stable asthma

**Figure 3:**
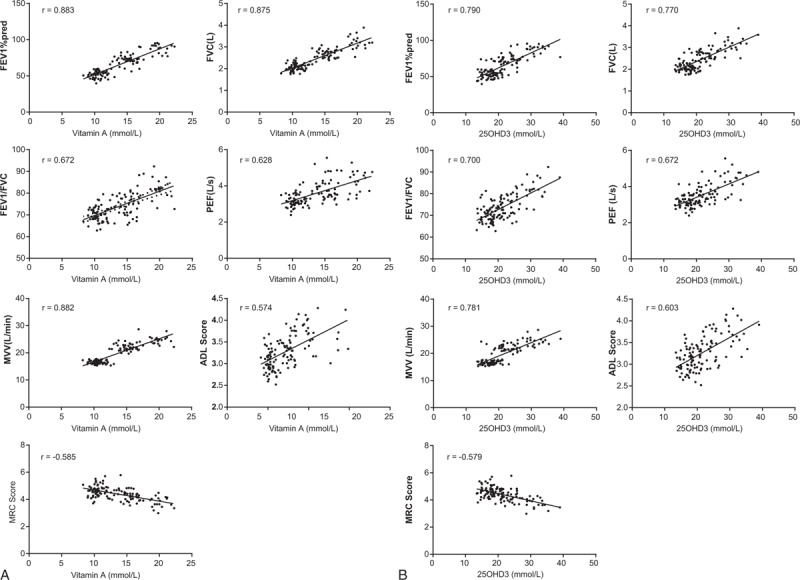
The serum vitamin A and 25OHD3 levels with pulmonary function and QOL. (A) Correlation of vitamin A with pulmonary function and quality of life; (B) correlation of 25OHD3 with pulmonary function and quality of life. ADL = activity of daily living, FEV1%pred = percentage of forced expiratory volume in 1 sec/predictive value, FVC = forced vital capacity, MRC = Medical Research Council, MVV = maximal voluntary ventilation, 25OHD3 = 25-hydroxyvitamin D3, PEF = peak expiratory flow, QOL = quality of life.

Pearson correlation analysis showed that serum vitamin A and 25OHD3 levels were positively correlated with pulmonary function indexes (FEV_1_%pred, FVC, FEV_1_/FVC, peak expiratory flow, and maximal voluntary ventilation) and ADL score in children with stable asthma, while serum vitamin A and 25OHD3 levels were negatively correlated with MRC score (all *P* < .05) (Table 4, Fig. 3).

**Table 4:**
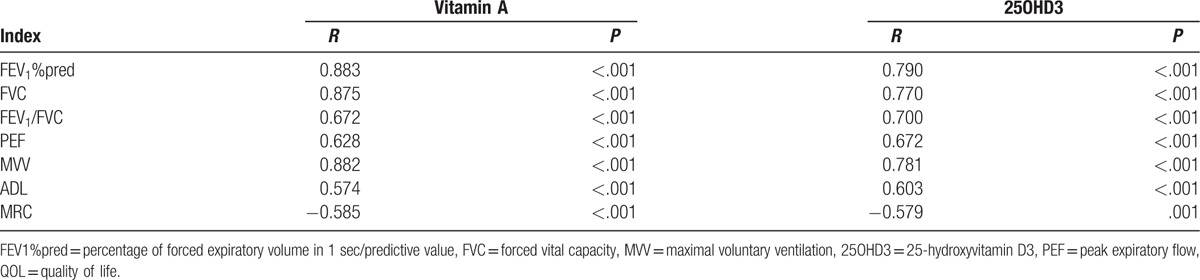
Correlations of the serum vitamin A and 25OHD3 levels with pulmonary function and QOL in children with stable asthma.

## 4 Discussion

In this study, we explored the relationship between different levels of serum vitamin A and 25OHD3 with pulmonary function and QOL in children with stable asthma. Our findings have demonstrated that increased serum vitamin A and 25OHD3 levels are correlated with good pulmonary function and a high QOL in children who are affected.

Results first showed that the serum calcium and phosphorus levels of children with stable asthma in the case group were lower than those in the healthy children control group. In line with our study, Oladipoo et al provided solid evidence that stable asthmatics have lower calcium and phosphorus levels compared to healthy controls.^[17]^ Interestingly, the result of correlation analysis showed that serum vitamin A and 25OHD3 levels were positively correlated with serum calcium and phosphorus levels. A relevant study demonstrated that Vitamin D is essential in serum calcium and phosphorus regulation, thereby explaining how high levels of vitamin D reflect proportional levels of serum calcium and phosphorous.^[18]^ Based on these findings, we can infer that vitamin A also plays a critical role in serum calcium and phosphorus regulation. 25OHD3 is associated positively with serum calcium and phosphorus, which is consistent with our study.^[19]^ The characteristic of asthma is airway hyper-responsiveness and variable airflow obstruction, partly caused by excessive contraction of airway smooth muscle, adhering to primary cell culture.^[20]^ A previous study also revealed that children with asthma had lower serum vitamin D compared to the healthy children; with vitamin D deficiency, the asthmatics also had lower phosphorus levels because of less exposure to sunlight and less physical activity.^[21]^

In our investigation, we found that the serum vitamin A and 25OHD3 levels in children with asthma were also lower than those in healthy children. Vitamin A plays an important role in the regulation of early lung development and alveolar formation,^[22]^ as well as the maintain of the immune system and needed for good vision.^[23]^ Vitamin A deficiency has been proven to result in an adverse effect on lung development and it is easy to suffer from asthma.^[24]^ One study suggested that serum vitamin A levels were lower in people with asthma than in healthy people.^[25]^ 25OHD3 is a known metabolite of vitamin D. Measuring the concentration of 25OHD3 is a useful tool to help determine the patient's vitamin D status.^[26]^ Research has shown that the level of vitamin D was found to be lower in patients with asthma when compared with the controls.^[27,28]^ It was suggested that vitamins A, D, and E, and antimicrobial peptide LL-37 had immunomodulatory effects, especially, serum vitamin A and 25OHD3 were associated with allergic and immune response, and higher vitamin A levels were related to less viral detection and higher 25OHD3 levels were associated with less allergic rhinitis and atopy.^[29]^ A study showed that 25OHD3 has antiinflammatory effect in Th2-dependent asthma model and 25OHD3 regulates the migration of cells and inhibits Th function at sites of inflammation, thus attenuating the inflammatory pathway in asthmatic conditions.^[30]^ Consistent with our results, previous findings also indicated that 25OHD3 levels were also lower in asthmatic patients compared to healthy people in spite of their allergy and increased asthma risk.^[31]^ In addition, a previous study revealed that decreased 25OHD3 level was associated with vitamin D deficiency,^[32]^ and children develop asthma related to vitamin D intake during pregnancy, and one study proves that higher vitamin D levels always accompany with decreasing severity of asthma,^[33]^ from which we could infer that decreased 25OHD3 level was related to asthma. Furthermore, another study showed that lower vitamin A level contributes to severity of asthma.^[34]^

Based on our pulmonary function measurements, we infer from our data that serum vitamin A/25OHD3 levels and lung function indexes decreased proportionally in different degrees of children with stable asthma according to the degree of asthma. Previous evidence revealed that children who had a predisposing condition of asthma had higher risk for developing a more moderate and severe form asthma.^[21]^ A study focused on serum 25OHD3 level and pulmonary function in patients with different phenotypes of asthma found that serum 25OHD3 level was associated with pulmonary function.^[31]^ Furthermore, our study illustrated that the QOL in children with asthma in the mild, moderate, and severe groups decreased obviously compared to the control group, with the severe group being the lowest. The QOL was highest in children in the mild group. QOL in patients with asthma is influenced by many factors: the severity of clinical symptoms, morbidity, gender, and the psychological resources available so as to deal with such difficulties.^[35]^ The asthma-specific QOL is a well-practical tool which could indicate the characteristic of patients with asthma and the QOL measured by it was more sensitive.^[36]^ Elkholy et al also confirmed that asthma could decrease health-related QOL.^[37]^ Pearson correlation analysis also confirmed that serum vitamin A and 25OHD3 levels were positively correlated with pulmonary function index and ADL score in children with stable asthma, while negatively correlated with MRC score.

In conclusion, our study demonstrated that increased serum vitamin A and 25OHD3 levels were related to good pulmonary function and good QOL in children with stable asthma. Although this study did not provide enough supporting evidence on how serum vitamin A and 25OHD3 can directly affect the onset of asthma, we thus need to rely on further large-scale trails to help draw other conclusions.

## References

[1] Wang Z Zhang H Sun X The protective role of vitamin D3 in a murine model of asthma via the suppression of TGF-beta/Smad signaling and activation of the Nrf2/HO-1 pathway. Mol Med Rep 2016;14:2389–96. 27484042 10.3892/mmr.2016.5563 PMC4991747

[2] Abu-Shaheen AK Nofal A Heena H Parental perceptions and practices toward childhood asthma. Biomed Res Int 2016;2016:6364194. 27843948 10.1155/2016/6364194 PMC5097792

[3] Turner S Predicting and reducing risk of exacerbations in children with asthma in the primary care setting: current perspectives. Pragmat Obs Res 2016;7:33–9. 27822136 10.2147/POR.S98928 PMC5087819

[4] Stoloff SW Asthma management and prevention: current perspectives. Clin Cornerstone 2008;9:6–20. discussion 21-3. 19410159 10.1016/s1098-3597(09)62036-6

[5] Li S Wang Y Shi Y Regulatory effects of stage-treatment with established Chinese herbal formulas on inflammatory mediators in pediatric asthma. J Tradit Chin Med 2013;33:727–32. 24660603 10.1016/s0254-6272(14)60004-2

[6] Dimov VV Stokes JR Casale TB Immunomodulators in asthma therapy. Curr Allergy Asthma Rep 2009;9:475–83. 19814921 10.1007/s11882-009-0070-x

[7] Bodzenta-Lukaszyk A Fal AM Jassem E The statement of the Polish Society of Allergology experts on the treatment of difficult-to-treat asthma. Pneumonol Alergol Pol 2015;83:324–34. 26166794 10.5603/PiAP.2015.0052

[8] Green AS Fascetti AJ Meeting the vitamin A requirement: the efficacy and importance of beta-carotene in animal species. ScientificWorldJournal 2016;2016:7393620. 27833936 10.1155/2016/7393620 PMC5090096

[9] Marquez HA Cardoso WV Vitamin A-retinoid signaling in pulmonary development and disease. Mol Cell Pediatr 2016;3:28. 27480876 10.1186/s40348-016-0054-6 PMC4969253

[10] Adela R Borkar RM Bhandi MM Lower vitamin D metabolites levels were associated with increased coronary artery diseases in type 2 diabetes patients in India. Sci Rep 2016;6:37593. 27883024 10.1038/srep37593 PMC5121614

[11] Lan N Luo G Yang X 25-Hydroxyvitamin D3-deficiency enhances oxidative stress and corticosteroid resistance in severe asthma exacerbation. PLoS One 2014;9:e111599. 25380286 10.1371/journal.pone.0111599 PMC4224414

[12] Rajanandh MG Nageswari AD Prathiksha G Effectiveness of vitamin D3 in severe persistent asthmatic patients: a double blind, randomized, clinical study. J Pharmacol Pharmacother 2015;6:142–6. 26311997 10.4103/0976-500X.162022 PMC4544135

[13] National Asthma E Prevention P Expert Panel Report 3 (EPR-3): Guidelines for the Diagnosis and Management of Asthma-Summary Report 2007. J Allergy Clin Immunol 2007;120:S94–138. 17983880 10.1016/j.jaci.2007.09.043

[14] Liu JM Hu HC Shi MH [The significance of volumetric capnography in assessment of asthmatic acute exacerbation staging]. Zhonghua Jie He He Hu Xi Za Zhi 2008;31:186–90. 18785516

[15] Kato T Kato Z Kuratsubo I Evaluation of adl in patients with hunter disease using fim score. Brain Dev 2007;29:298–305. 17307320 10.1016/j.braindev.2006.08.015

[16] Eltayara L Becklake MR Volta CA Relationship between chronic dyspnea and expiratory flow limitation in patients with chronic obstructive pulmonary disease. Am J Respir Crit Care Med 1996;154:1726–34. 8970362 10.1164/ajrccm.154.6.8970362

[17] Oladipo OO Chukwu CC Ajala MO Plasma magnesium in adult asthmatics at the lagos university teaching hospital, Nigeria. East Afr Med J 2003;80:488–91. 14640172 10.4314/eamj.v80i9.8748

[18] Pozza ME Kaewsakhorn T Trinarong C Serum vitamin D, calcium, and phosphorus concentrations in ponies, horses and foals from the United States and Thailand. Vet J 2014;199:451–6. 24524849 10.1016/j.tvjl.2014.01.002

[19] Van Damme P Robberecht W Clinical implications of recent breakthroughs in amyotrophic lateral sclerosis. Curr Opin Neurol 2013;26:466–72. 23945281 10.1097/WCO.0b013e328364c063

[20] Singh SR Sutcliffe A Kaur D CCL2 release by airway smooth muscle is increased in asthma and promotes fibrocyte migration. Allergy 2014;69:1189–97. 24931417 10.1111/all.12444 PMC4215601

[21] Bener A Ehlayel MS Tulic MK Vitamin D deficiency as a strong predictor of asthma in children. Int Arch Allergy Immunol 2012;157:168–75. 21986034 10.1159/000323941

[22] Checkley W West KP Jr Wise RA Maternal vitamin A supplementation and lung function in offspring. N Engl J Med 2010;362:1784–94. 20463338 10.1056/NEJMoa0907441

[23] Tanumihardjo SA Vitamin A: biomarkers of nutrition for development. Am J Clin Nutr 2011;94:658S–65S. 21715511 10.3945/ajcn.110.005777 PMC3142734

[24] Checkley W West KP Jr Wise RA Supplementation with vitamin A early in life and subsequent risk of asthma. Eur Respir J 2011;38:1310–9. 21700611 10.1183/09031936.00006911 PMC7305825

[25] Al Senaidy AM Serum vitamin A and beta-carotene levels in children with asthma. J Asthma 2009;46:699–702. 19728208 10.1080/02770900903056195

[26] Holick MF DeLuca HF Avioli LV Isolation and identification of 25-hydroxycholecalciferol from human plasma. Arch Intern Med 1972;129:56–61. 4332591

[27] Ocampo-Pelland AS Gastonguay MR French JF Model-based meta-analysis for development of a population-pharmacokinetic (PPK) model for Vitamin D3 and its 25OHD3 metabolite using both individual and arm-level data. J Pharmacokinet Pharmacodyn 2016;43:191–206. 26872884 10.1007/s10928-016-9465-1

[28] Hatami G Ghasemi K Motamed N Relationship between vitamin D and childhood asthma: a case-control study. Iran J Pediatr 2014;24:710–4. 26019776 PMC4442832

[29] Elenius V Palomares O Waris M The relationship of serum vitamins a, d, e and ll-37 levels with allergic status, tonsillar virus detection and immune response. PLoS One 2017;12:e0172350. 28235040 10.1371/journal.pone.0172350 PMC5325266

[30] Topilski I Flaishon L Naveh Y The anti-inflammatory effects of 1,25-dihydroxyvitamin D3 on Th2 cells in vivo are due in part to the control of integrin-mediated T lymphocyte homing. Eur J Immunol 2004;34:1068–76. 15048717 10.1002/eji.200324532

[31] Tamasauskiene L Gasiuniene E Lavinskiene S Evaluation of vitamin D levels in allergic and non-allergic asthma. Medicina (Kaunas) 2015;51:321–7. 26739673 10.1016/j.medici.2015.11.003

[32] Yu S Fang H Han J The high prevalence of hypovitaminosis d in china: a multicenter vitamin d status survey. Medicine (Baltimore) 2015;94:e585. 25715263 10.1097/MD.0000000000000585 PMC4554140

[33] Brehm JM Celedon JC Soto-Quiros ME Serum vitamin D levels and markers of severity of childhood asthma in Costa Rica. Am J Respir Crit Care Med 2009;179:765–71. 19179486 10.1164/rccm.200808-1361OC PMC2675563

[34] Arora P Kumar V Batra S Vitamin a status in children with asthma. Pediatr Allergy Immunol 2002;13:223–6. 12144646 10.1034/j.1399-3038.2002.00010.x

[35] Alvim CG Picinin IM Camargos PM Quality of life in asthmatic adolescents: an overall evaluation of disease control. J Asthma 2009;46:186–90. 19253128 10.1080/02770900802604129

[36] Lee EH Kim SH Choi JH Development and evaluation of an Asthma-Specific Quality of Life (A-QOL) questionnaire. J Asthma 2009;46:716–21. 19728212 10.1080/02770900903067887

[37] Holland AC Kensinger EA An fMRI investigation of the cognitive reappraisal of negative memories. Neuropsychologia 2013;51:2389–400. 23500898 10.1016/j.neuropsychologia.2013.02.012 PMC3723712

